# Impact of endurance exercise and probiotic supplementation on the intestinal microbiota: a cross-over pilot study

**DOI:** 10.1186/s40814-019-0459-9

**Published:** 2019-06-08

**Authors:** Laura Schmitz, Nina Ferrari, Andreas Schwiertz, Kerstin Rusch, Ulrich Woestmann, Esther Mahabir, Christine Graf

**Affiliations:** 1 Institute of Movement and Neuroscience, Department of Movement and Health Promotion, German Sport University Cologne, Am Sportpark Müngersdorf 6, Cologne, Germany; 2 MVZ Institute of Microecology, Auf den Lueppen 8, Herborn, Germany; 3 Academic postgraduate training practice of the University Düsseldorf, Düsseldorf, Germany; 4 Comparative Medicine, Center for Molecular Medicine, University of Cologne, Robert-Koch-Str. 21, Cologne, Germany; 5 Cologne Center for Prevention in Childhood and Youth/Heart Center Cologne, University Hospital of Cologne, Kerpener Str. 62, Cologne, Germany

**Keywords:** Physical activity, Cardiorespiratory fitness, Exercise, Intestinal microbiota, Intestinal health, Probiotics

## Abstract

**Background:**

The human microbiota has a broad range of functions contributing to metabolic processes and the activities of our immune system. Its influence on health, well-being and chronic diseases are discussed in various studies. The intestinal microbiota and the mucosal integrity are influenced by diet, environment and other lifestyle factors, including physical activity. There are correlations between cardiorespiratory fitness and important markers of intestinal health. However, data linking endurance exercise to microbiota composition are sparse. Many endurance athletes take probiotics to reduce gastrointestinal symptoms linked to exercise or immunosuppression, but the longitudinal data is insufficient.

This randomised, controlled cross-over pilot study will examine the impact of specific endurance training and probiotic supplementation on the intestinal microbiota and mucosa in healthy, athletic students.

**Objective:**

The aim of this pilot study is to elucidate the impact of physical activity on the intestinal microbiota and mucosa with regard to the effects of a probiotic supplementation.

**Methods:**

In this pilot study, thirty non-specifically trained student athletes will participate in an intervention consisting of a two-week rest (baseline) period, a four-week exercise programme and a four-week probiotic intervention using SymbioLactComp®. The exercise programme consists of three 60-min running workouts per week at 70–85% of the peak heart rate (HRpeak). Primary endpoint of this pilot study is the feasibility and practicality of the intervention as well as a sample size estimation. Furthermore, anthropometric measurements and information on nutrition and lifestyle will be obtained. The peak oxygen uptake (VO_2_peak) and peak heart rate (HRpeak) (determined during a shuttle run test) as well as selected blood and saliva parameters (haemogram, cytokines) will be evaluated. Changes to the intestinal microbiota will be analysed by stool diagnostics (KyberKompaktPRO®, KyberPlus®). The potential changes may include microbiota composition, bacterial metabolites and mucosa- and immune markers.

**Conclusion:**

Results will be used for the design of a main randomised controlled trial with a larger collective based on feasibility, validity and sample size estimation as well as the potential effects of endurance exercise on intestinal microbiota and mucosa. Evidence-based information of an exercise-altered microbiota could be of importance for the prevention and therapy of intestinal or immune disorders.

**Trial registration:**

German Clinical Trials Register: DRKS00011108. Retrospectively registered on 28 November 2016.

**Electronic supplementary material:**

The online version of this article (10.1186/s40814-019-0459-9) contains supplementary material, which is available to authorized users.

## Introduction

The intestinal microbiota composition plays a significant role in metabolism and immunity [1, 2]. Certain species and their associated metabolic products influence physiological homeostasis. For instance, the microbiota is involved in the degradation of food and the synthesis of vitamins, bioactive compounds and hormones such as serotonin. It further plays a role in the training of the immune system, pathogen defence and proliferation of intestinal cells [3, 4]. There is no recognised definition of an intact microbiota. However, the following aspects are believed to play an essential role: an enriched bacterial diversity (alpha diversity), a balanced *Firmicutes*/*Bacteroidetes* ratio, an intact mucus layer and an abundance of certain species (e.g. *Akkermansia muciniphila* and *Faecalibacterium prausnitzii*) [5–9]. An altered microbiota contributes to the pathogenesis of diseases such as metabolic or (auto)-immune diseases [10–13]. Recent studies have highlighted that environment and lifestyle factors affect the microbiota composition and its metabolic capacity [14, 15]. There is a growing body of evidence indicating that physical activity is linked to specific markers of intestinal health [16–19].

Several studies determined a higher species richness (*α* diversity) in elite athletes and individuals with higher cardiorespiratory fitness (VO_2_peak) or higher training frequency than those with a sedentary lifestyle or lower fitness level [16, 17, 20].

Furthermore, an increase in number of the two commensal species *Akkermansia muciniphila* and *Faecalibacterium prausnitzii* was observed in athletes and highly active individuals [17, 18, 20]. This was related to a higher concentration of the health-promoting bacterial metabolite butyrate [18, 20].

Some professional athletes suffer from immunosuppression or gastrointestinal symptoms, including abdominal pain, diarrhoea or leaky gut syndrome. This is because excessive exercise reduces intestinal blood flow due to increased circulation in the strained muscles and heart. This may lead to microbial imbalances and mucosa disruption [19, 21–24]. An increased permeability of the intestinal mucosa and epithelium (intestinal barrier) has been associated with bacterial/pathogen translocation to extraintestinal organs manifesting as intestinal or systemic inflammation [25–27].

Many athletes use probiotics to enhance barrier integrity and to improve gastrointestinal symptoms. The consumption of a probiotic supplement may induce immune processes, including the synthesis of cytokines and immunoglobulins, thereby contributing to the host’s health [28–30]. Previous research with trained athletes revealed the beneficial effects of probiotic intake on zonulin, a marker of mucosal integrity and several cytokines. Interferon-gamma, tumour necrosis factor-alpha and interleukins were all improved [19, 29, 31–33].

Regular moderate exercise is well-known for its anti-inflammatory effects, but diet may be a confounding factor in exercise-related impacts on the gastrointestinal system [17, 18]. Data that determines causality between microbiota composition, mucosa permeability and moderate endurance training is sparse. Longitudinal studies are needed to determine how the intestinal microbiota and mucosa as well as immune markers and cytokines vary with exercise as compared to probiotic supplementation. A combination of exercise and probiotics may be a new method for the prevention and therapy of intestinal or immune-related diseases [34–36].

### Research hypothesis

We hypothesise that a training intervention with regular running exercises may alter microbiota composition in healthy adults. Participants receiving the intervention might further show higher rates of selected markers of intestinal health such as *Akkermansia muciniphila, Faecalibacterium prausnitzii, Bifidobacterium* spp. and lower rates of intestinal immune markers, e.g. zonulin, alpha-1-antitrypsin or secretory immunoglobulin A (sIgA).

### Aims and objectives

Primary outcomes of this randomised, controlled, cross-over pilot trial are the feasibility and practicality of the intervention, the validity of the study design and the provision of a reliable estimate for sample size calculation. To assess the practicality of the intervention, the duration (60 min), frequency (three times per week) and intensity (70–80% HRpeak) of the exercise programme will be considered as well as the adherence to the daily probiotic supplementation. The validity of the study design will be assessed with regard to the collection and immediate shipping of faecal samples. A further assessment will be made considering the time commitment for participants to complete endurance training, anthropometric testing, faecal samples and food diaries. In order to evaluate the efficacy of the intervention, the influence of moderate endurance training compared to probiotic supplementation (SymbioLactComp®) on the intestinal microbiota composition and barrier function (selected mucosal and immune parameters) as well as on selected blood and saliva biomarkers (haemogram, selected cytokines) in healthy student athletes will be examined.

## Methods

### Pilot trial design, study setting and ethics

This randomised controlled pilot cross-over trial is conducted at the German Sport University Cologne in cooperation with the MVZ Institute of Microecology GmbH. Data will be collected through bachelor endurance courses at the German Sport University Cologne (Cologne, Germany).

The study was approved by the Ethics Committee of the German Sport University Cologne (No. 136/2016). A schematic of the study design is given in more detail (see Additional file 1). To ensure all necessary aspects were addressed in the study protocol, the Standard Protocol Items: Recommendations for Interventional Trials (SPIRIT) checklist was adhered [37, 38] (Additional file 2).

### Experimental design

Participants will receive a 12-week intervention programme including a 2-week rest period, a 4-week endurance training programme and a 4-week probiotic intervention with SymbioLactComp®. Subjects will not perform endurance sports or take probiotics during the rest period but will participate in the university courses during the programme (e.g. gymnastics, volleyball, tennis).

#### Exercise intervention

Exercise intensity will be calculated based on the HRpeak determined during a shuttle run test performed at the outset of the study. The training goal is a 10-km run in 50 min (males) or 55 min (female). The intensity of exercises will increase from 70% to 85% of the HRpeak to improve the subject’s basic endurance [39]. The three 60-min running sessions per week will consist of extensive and intensive continuous methods, interval training and stretching [39]. The exercises will be performed in groups and supervised by a sports scientist. Ratings of perceived exertion will be checked using a Borg Scale; the heart rate will be measured using a pulse monitor and belt from Polar® (Polar® Typ FT1). Participants will keep a training diary to log the completion of exercises. A training plan is shown in more detail in the appendix (see Additional file 3).

#### Probiotic intervention

The probiotic period involves supplementation with the probiotic SymbioLactComp® (SymbioPharm GmbH, Herborn, Germany). SymbioLactComp® contains different lactic acid bacteria (*Lactobacillus paracasei*, *Lactobacillus acidophilus*, *Lactococcus lactis* and *Bifidobacterium animalis* subsp. *lactis*) as well as 30 μg of biotin. The total bacteria count of one sachet (2 g) is ≥ 1 × 10^9^ KBE/g. One sachet will be mixed with water and taken daily at breakfast.

### Pre-experimental procedures

#### Anthropometry

Anthropometric characteristics, arterial blood pressure and body composition will be measured. Body weight will be determined barefoot and in light clothes using a calibrated digital scale (Seca® Typ 771). Body height will be measured by a wall-mounted measuring tape (Seca® Typ 225). Body mass index will be calculated by the World Health Organization’s specifications (BMI = body weight (kg)/body size (m)^2^) [40]. Systolic and diastolic blood pressures will be measured three times after 5 min of sitting calmly using a digital blood pressure monitor (OMRON® Typ M5-1). Body fat and muscle mass will be estimated using bioelectric impedance analysis (EGOFIT GmbH, Eggstätt, Germany).

Furthermore, information on the participant’s personal history and lifestyle including physical activity levels will be collected prior to the start of the trial (T0) using a modified questionnaire (International Physical Activity Questionnaire; [41].

#### Cardiorespiratory fitness

A standardised multistage 20-m shuttle run test will be performed at the outset of the study to calculate HRpeak and the VO_2_peak as an indicator of the participants’ cardiorespiratory fitness [42].

The test will be executed by running back and forth on a 20-m course. A sound signal emitted from a prerecorded tape will set the pace. The starting speed is 8.5 km/h and will be increased by 0.5 km/h each minute, representing a stage. Participants must touch the 20-m line even after the acoustic signal. If the participant is not able to follow the pace, the test is finished. The last stage number will be used to determine the HRpeak by a pulse monitor and belt (Polar® Typ FT1) as well as the VO_2_peak. VO_2_peak will be calculated according to Léger and Lambert [42]: VO_2_max (mL kg^−1^ min^−1^) = − 24.4 + 6.0 × “maximal shuttle run speed”/km h^−1^. The shuttle run test will be repeated after the 4-week training period in order to define the potential performance improvement.

### Data collection

#### Blood samples

Venous blood samples (of approximately 25 mL) will be collected under medical supervision using the BD Vacutainer® Butterfly Safety Lok, EDTA tubes and SST™ II Advance serum tubes (Becton Dickinson, Heidelberg, Germany). For the evaluation of the chosen parameters, participants are not required to be fasted. A haemogram will be measured from the EDTA blood using a Sysmex KX-21N automated haematology analyser (Sysmex Deutschland GmbH, Bornbach, Germany). Two serum samples will be centrifuged at 4000×*g* for 10 min and pipetted and stored at − 80 °C in the Center for Molecular Medicine Cologne (University of Cologne, Germany) until it will be analysed. The thawed serum samples will be used to measure the following cytokines by multiplex ELISA (Bio-Plex Pro Human Cytokine 8-plex ELISA Kit, Bio-Plex Pro Human Chemokine IL-1β Singleplex Set; Bio-Rad Laboratories GmbH, Munich, Germany): tumour necrosis factor-alpha, interleukin-6, interleukin-8 and interleukin-1β.

#### Saliva samples

Saliva samples will be collected after a 30-min fast via Salivettes® from Sarstedt (Sarstedt AG & Co. KG, Nümbrecht, Germany). Samples will be centrifuged at 4000×*g* for 10 min and pipetted and stored at − 80 °C in the Center for Molecular Medicine Cologne (University of Cologne, Germany). The following selected cytokines will be analysed by a multiplex ELISA (Bio-Plex Pro Human Cytokine 8-plex, Bio-Rad Laboratories GmbH, Munich, Germany): tumour necrosis factor-alpha, interleukin-6, interleukin-8 and interferon-gamma.

#### Faecal samples

Faecal analyses will be carried out at the Institute of Microoecology, Herborn, Germany. Faecal samples will be collected during the final day of the dietary programme. Subjects will be given a sterile stool sampling kit and detailed sample collection instructions. Subjects will send samples to the laboratory, and if shipping is not possible immediately after collection, the sample will be stored in a refrigerator at 7 °C but will be sent on the same day.

#### Nutritional data

Each participant will complete four detailed 7-day food diaries based on self-reported intake prior to the next faecal sample collection. A standardised protocol will be developed by a research dietitian supervisor. Subjects will maintain a habitual diet, such that potential gastrointestinal modifications can only be attributed to the intervention of sports or probiotics.

Nutritional quality and quantity of consumed foods and drinks as well as nutritional supplements will be recorded. A prepared protocol with instructions will help to guide the data recorded: breakfast, lunch, dinner, snacks, recipe information, preparation method (raw, steamed, boiled, fried, breaded, baked, with/without skin), fat/fructose content and food amount (gramme/millilitre).

Dietary data and personal data (gender, age, body height, body weight, body mass index) will be entered into the DGExpert software (version 1.8.9.0) to generate each subject’s energy and nutrient intake as well as respective reference values.

The following list of dietary parameters will be analysed each day in grammes/microgrammes/percentage:

- Energy/caloric intake (kilocalories, kilojoule)
- Proteins
- Fats
- Total fatty acids
- Polyunsaturated fatty acids
- Monounsaturated fatty acids
- Saturated fatty acids
- Linoleic acid
- Linolenic acid
- Quotient of linoleic acid to linolenic acid
- Total carbohydrates
- Absorbable carbohydrates
- Fibres
- Lactose
- Sucrose
- Monosaccharides
- Disaccharides
- Total sugar
- All vitamins and micronutrients
- Water
- Alcohol

### Laboratory procedures

#### Identification and enumeration of microorganisms

Bacteria will be enumerated using the KyberKompaktPro® test, which combines the identification of viable bacteria by classical microbial analysis and quantitative polymerase chain reaction (qPCR).

Viable bacteria will be enumerated on the following selective media: Columbia blood agar (total cell count; BioMérieux, Nürtingen, Germany), U3G agar (*Enterobacteriacae*, *enterococci*; Heipha, Heidelberg, Germany), Rogosa agar, (*Lactobacilli*; Heipha), DIC agar (*Bifidobacteria*; Heipha), Schaedler agar (*Bacteroides*; Heipha) and SPM agar (*Clostridia*; Heipha). Faecal samples will be serially diluted in 1 mL of phosphate-buffered saline (PBS, pH 7.2) and subsequently plated on selective agar plates by a fully automated spiral plater (PreviIsola, BioMérieux). The plates will be incubated under either aerobic or anoxic conditions at 37 °C for at least 2 days. Bacteria will be identified by gram staining and colony morphologies. Additionally, identifications will be performed by the API and VITEK systems (BioMérieux). All counts will be recorded by the number of log_10_ CFU per grammes of sample.

The following bacteria will be routinely analysed: *Clostridium* spp., *Bifidobacterium* spp., *Bacteroides* spp., (*E. coli*, *Enterococcus* spp., *Lactobacillus* spp.) and other bacteria (*Pseudomonas* spp., *Klebsiella* spp*.*, *Proteus* spp., *Citrobacter* spp*., Enterobacter* spp., other aerobic bacteria).

#### DNA extraction from faecal samples for further qPCR analysis

Microbial DNA will be extracted from 200 mg of faecal samples using the QIAsymphony® DSP Virus/Pathogen Mini-Kit (Qiagen, Hilden, Germany) following the manufacturer’s instructions on the QIAsymphony® SP (Qiagen). Automated isolation and pipetting of 96-well plates (MicroAmp Optical 96-Well Reaction Plate, Applied Biosystems, Darmstadt, Germany) will be performed by the QIAsymphony® SP/AS instrument (Qiagen) using the QIAsymphony DSP Virus/Pathogen Mini-Kit.

#### Quantification of target bacteria by quantitative real-time PCR (qPCR)

Primers will be selected to recognise the phyla *Firmicutes*, *Bacteroidetes* [43], *Verrucomicrobia*, the genus *Enterobacteriacea* [44] and *Methanobrevibacter* as well as the species *Faecalibacterium prausnitzii* and *Akkermansia muciniphila*.

PCR amplification and detection will be performed using an ABI PRISM 7900HT sequence detection system (Applied Biosystems, Darmstadt, Germany) in optical-grade 96-well plates sealed with optical sealing tape. Each reaction mixture (25 μL) will be composed of 12.5 μL of QuantiTect SYBR Green PCR Master Mix (Qiagen), 2 μL primer mix (10 pmol/μL each), 9 μL sterile distilled H_2_O and 1.5 μL stool DNA (10 ng/μL). For the negative control, 2 μL of sterile distilled H_2_O will be added to the reaction solution instead of the template DNA solution. A standard curve will be produced using the appropriate reference organism to quantify the qPCR values into the number of bacteria per grammes. The standard curves will be prepared in the same PCR assay as the samples. The fluorescent products will be detected in the last step of each cycle. A melting curve analysis will be carried out following amplification to distinguish the targeted PCR product from the non-targeted PCR product. The melting curves will be obtained by slowly increasing the temperature to 55 and 95 °C at a rate of 0.2 °C/s, with continuous fluorescence collection. The data will be analysed using ABI Prism software. The real-time PCRs will be performed in triplicate, and average values will be used for enumeration.

#### Laboratory analyses of short-chain fatty acids

Human stool samples analysed for short-chain fatty acid (SCFA) content will be freeze-dried and subsequently analysed using a gas chromatograph. The samples will be weighed (~ 200 mg of dry matter) and a 10-fold dilution with physiological NaCl saline (1.8 mL) will be produced. Following vortexing (2 min, Vortexer MS 3 basic, IKA-Werke GmbH & Co. KG, Staufen, Germany), an aliquot of 200 μL will be mixed with 0.36 M HClO_4_ (280 μL) and 1 M NaOH (270 μL). The solution will be lyophilised at − 35 °C (alpha 1–4 LSC, CHRIST, Osterode am Harz, Germany). The obtained lyophilisate will be dissolved in 100 μL 5 M HCOOH and 400 μL acetone and centrifuged (5 min at 4000×*g*, RT; Pico 17, Thermo Electron LED GmbH, Langenselbold, Germany). Concentrations of the SCFA will be determined in the supernatant using a GC-2010 Plus gas chromatograph (Shimadzu Deutschland GmbH, Duisburg, Germany) equipped with flame ionisation detection and a thin-film capillary column Stabilwax®-DA 30 m × 0.25 mm × 0.5 μm (Restek, Bad Homburg, Germany). The samples will be spread out by split injection using the auto-sampler AOC-20s/I (Shimadzu Deutschland GmbH). GCsolution Chromatography Data System (Shimadzu Deutschland GmbH) will be used for data processing. For the determination of the SCFA, an external standard (Supelco™ WSFA-1 Mix, Supelco Sigma-Aldrich Co., Bellefonte PA) will be used.

#### Laboratory analyses of faecal markers

Faecal calprotectin, eosinophil protein x (EPX), zonulin, beta-defensin 2 (β-defensin 2) and secretory immunoglobulin A (sIgA) concentrations will be measured by an ELISA kit (Immundiagnostik AG, Bensheim, Germany). The tests to analyse calprotectin and EPX use the sandwich ELISA technique. Monoclonal anti-calprotectin-antibodies or anti-EPX-antibodies will be added to the samples, standards and controls. Colour intensity is directly proportional to the concentration of calprotectin and EPX. Samples will be read at 450 nm. Standard curves will be formed by the four-parameter algorithm.

A competitive binding technique will be used to determine zonulin concentrations. Samples, standards and controls will be added with biotinylated zonulin and incubated with polyclonal anti-zonulin antibodies. Colour intensity is inversely proportional to the sample concentration of zonulin. Samples will be read at 450 nm. The four-parameter algorithm will be used to create the standard curve [45].

Quantitative analysis of β-defensin 2, standards and controls will be performed using polyclonal anti-β-defensin 2 antibodies. The amount of bound enzymes is directly proportional to the β-defensin 2 content. Samples will also be measured at 450 nm.

To determine secretory immunoglobulin A (sIgA), polyclonal anti-sIgA antibodies will be added to the samples. The test uses a calibrator and calibration curve to detect sIgA content. Colour development is proportional to the amount of analyte.

Faecal alpha-1-antitrypsin concentrations will be measured using the AAT test (Maier Analytic, Sinsheim, Germany). The assay uses the sandwich technique and polyclonal antibodies will be utilised to analyse samples, standards and controls. Colour intensity is directly proportional to the alpha-1-antytrypsin concentration in the sample. The samples will be read at 450 nm and the standard curve formed via the four-parameter algorithm.

### Sample size

It is estimated that a sample size of a minimum of 30 participants is required in order to provide a pooled standard deviation that provides enough precision for the development of a future trial [46, 47].

### Participants and recruitment

The study will have a half-year enrollment phase. Recruitment will be carried out by the study director. Healthy students with an age between 18 and 29 and a body mass index of ≤ 25 kg/m^2^ will be recruited through bachelor endurance courses at the German Sport University Cologne (maximal number of participants around 320 students). Therefore, our feasibility study enrollment rate has to exceed 10%. The recruitment rate will be analysed by the number of eligible individuals recruited within half a year, as presented above. A recruitment rate of > 80% will be considered as feasible to conduct a future randomised control trial.

### Randomisation

Participants will be randomised in a 1:1 ratio and allocated to group one (endurance-probiotics) or group two (probiotics-endurance) via block randomisation by the study director [48]. Exclusion criteria will be injuries; diseases (e.g. inflammatory bowel diseases), an antibiotic therapy just before or during the intervention; and continuing gastrointestinal disturbances based on probiotic intake. Subjects who meet the inclusion criteria will obtain detailed information about the trial and will give written informed consent before starting the study.

### Statistical analysis

This pilot study intends to examine the confidence interval estimation and feasibility of the presented approach (physical activity and probiotic supplementation) [49, 50]. Attendance and retention rates will be assessed to calculate adherence. Attendance rate will be analysed by dividing the mean value for the number of participants present by the number of sessions offered during the four-week exercise period and the four-week probiotic intervention. The retention rate will be analysed by the percentage of participants completing all sessions of the endurance training (twelve in total), shuttle run test (two in total), nutrition questionnaire (four in total), blood/saliva measurement (four in total) and stool diagnostics (four in total) present in each step of the study. A retention rate of > 80% will be considered as feasible to conduct a future randomised controlled trial. Hypothesis testing and results of regression modelling will be considered entirely exploratory in nature and interpreted with caution. Our trial is mainly conducted in preparation for a future definitive randomised controlled trial to assess the efficacy of the intervention/study design [51]. All data will be entered into the database management software and analysed by the SPSS Version 25.0 (SPSS Inc., Chicago, IL, USA). The Shapiro-Wilk test will be used for variable normal distribution and the usual descriptive statistics for background information and mean values with a 95% confidence interval. Data will be used for sample size estimation and to determine the most appropriate outcome measure for the main study. Descriptive statistics and confidence intervals will also be calculated to examine the trends in the analysed parameters. This information will help to identify which parameters to focus on in future trials. Depending on the normality of the underlying data, parametric or non-parametric (e.g. unpaired *t* test/Mann-Whitney *U* test) tests will be carried out. Furthermore, a paired *t* test/Wilcoxon test might be computed for the longitudinal comparison (T0–T3). Bivariate correlation analyses might be assessed by the Spearman/Pearson correlation coefficient. Multivariate analyses with linear or logistic regressions might be performed to explore potential effects of the intervention and further potential covariates and cofactors. Moreover, multiple regression models might be used if multiple variables are predictive. The level of statistical significance is *p* < 0.05.

## Discussion

Several cross-sectional studies have determined microbial differences between people living in an active or sedentary lifestyle. However, there is a lack of investigations exploring the effects of sport independent of diet [16–18]. By comparing a controlled exercise programme to probiotic supplementation (cross-over design) and including nutritional analyses, this pilot trial intends to examine a causal relationship in this complex system. Progression criteria were described to support the concept and the effectiveness of the intervention in order to analyse the outcome parameters in the future. The use of the progression criteria intends to validate the plausibility and clinical importance of this kind of intervention, which can serve as the basis for a main randomised controlled trial. This approach is of importance because evidence-based information on exercise-altered microbiota is needed for the prevention and therapy of intestinal or immune disorders.

## Additional files

Additional file 1: Study design (PDF 1470 kb)

Additional file 2: SPIRIT checklist (PDF 243 kb)

Additional file 3: Training plan (PDF 185 kb)

## Abbreviations

EPX: Eosinophil protein x
HRpeak: Peak heart rate
IPAQ: International Physical Activity Questionnaire
PCR: Polymerase chain reaction
SCFAs: Short-chain fatty acids
sIgA: Secretory immunoglobulin A
VO_2_peak: Peak oxygen uptake
β-defensin 2: beta-defensin 2
