# Supplementary material for: Impact of endurance exercise and probiotic supplementation on the intestinal microbiota: a cross-over pilot study
Source: Pilot Feasibility Stud. 2019 Jun 8;5:76. doi: 10.1186/s40814-019-0459-9 (PMC6556026; doi:10.1186/s40814-019-0459-9)
Supplement: Supplementary file 1 — Study design (PDF 1470 kb) [file 40814_2019_459_MOESM1_ESM.pdf]

Recruitment of 30 healthy athletic students at
German Sport University Cologne
Randomisation of participated subjects into two intervention groups

Intervention (T0, T1, T2, T3):

Microbiota analysis

(Escherichia coli, E. coli biovare, Proteus spp., Klebsiella spp., Pseudomonas spp., Enterobacter spp., Citrobacter spp., Enterococcus spp., Bifidobacterium spp., Bacteroides spp., Lactobacillus spp., H_2O_2 Lactobacilli, Clostridium spp., Feacalibacterium prausnitzii, Akkermansia muciniphila, yeasts, mold, total bacterial count, fecal pH-value, secretory immunoglobulin A, eosinophile protein X, β-Defensin 2, zonulin, alpha 1-antitrypsin, calprotectin, butyrate)

Blood analysis

(leucocytes, erythrocytes, haemoglobin, hematocrit, thrombocytes, mean corpuscular volume, tumor necrosis factor-alpha, interleukin-6, interleukin-8, interleukin-1β)

Saliva analysis

(tumor necrosis factor-alpha, interleukin-6, interleukin-8, interferon-gamma)

Nutritional analysis

(calories, carbohydrates, fats, proteins, fibres, vitamins)

Anthropometry

(height, weight, blood pressure, body fat, muscle mass)

20 m shuttle run test

(peak oxygen uptake, peak heart rate)

Lifestyle questionnaire

(physical activity, lifestyle data)

2-week resting period
Baseline

group 1

4-week exercise period

3 workouts per week

group 2

4-week probiotic period

daily intake

4-week exercise period

3 workouts per week

4-week probiotic period

daily intake

Additional file shows study design
