# Supplementary material for: Impact of endurance exercise and probiotic supplementation on the intestinal microbiota: a cross-over pilot study
Source: Pilot Feasibility Stud. 2019 Jun 8;5:76. doi: 10.1186/s40814-019-0459-9 (PMC6556026; doi:10.1186/s40814-019-0459-9)
Supplement: Supplementary file 2 — SPIRIT checklist (PDF 243 kb) [file 40814_2019_459_MOESM2_ESM.pdf]

SPIRIT
STANDARD PROTOCOL ITEMS: RECOMMENDATIONS FOR INTERVENTIONAL TRIALS

SPIRIT 2013 Checklist: Recommended items to address in a clinical trial protocol and related documents*

| Section/item | Item No | Description |
|---|---|---|
| **Administrative information** | | |
| Title | 1 | Impact of endurance exercise and probiotic supplement on the intestinal microbiota; a study protocol |
| Trial registration | 2a | German Clinical Trials Register: DRKS00011108. Retrospectively registered on 28 November 2016. |
| | 2b | DRKS-ID: **DRKS00011108**<br>Sources of monetary or material support:<br>MVZ Institute of Microecology, Herborn, Germany.<br>Primary sponsor:<br>MVZ Institute of Microecology, Herborn, Germany.<br>Contact of public queries:<br>Laura Schmitz, l.schmitz@dshs-koeln.de, tel: 0221 4982 5290.<br>Contact of scientific queries:<br>Laura Schmitz, l.schmitz@dshs-koeln.de, Institute of Movement and Neuroscience, Department of Movement and Health Promotion, Am Sportpark Müngersdorf 6, German Sport University Cologne, Cologne, Germany (tel: 0221 4982 5290).<br>Public title: Impact of endurance exercise and probiotic supplement on the intestinal microbiota; a study protocol<br>Country of recruitment: Cologne, Germany<br>Interventions:<br>Active comparator: endurance training (3 times/ week)<br>Placebo comparator: probiotic SymbioLactComp®<br>Key inclusion criteria: written consent, age between 18 and 29, medical certificate to check and prove sport apitude.<br>Key exclusion criteria: injuries that do not allow participation in the endurance program, chronic diseases, especially intestinal diseases, eating disorders, regular medication.<br>Study type: interventional<br>allocation: controlled randomised study<br>Intervention model: cross-over design; block randomisation<br>Primary purpose: prevention<br>Target sample size: 30<br>Recruitment status: recruiting<br>Primary outcomes: intestinal microbiota composition, intestinal immune- and mucosa markers (selected microbial genera, short chain fatty acids, calprotectin, eosinophil protein x, zonulin, beta-defensin-2, secretory immunoglobulin A.<br>Key secondary outcomes: selected blood and saliva markers (haemogram, tumor necrosis factor-alpha, interleukin-6, interleukin-8, interleukin-1β, interferon-gamma). |

| | | |
|---|---|---|
| Protocol version | 3 | Manuscript number: PAFS-D-18-00101<br>Revision Chronology:<br>2018/06/15 - Original; Initial Date submitted<br>2019/01/10 - Amendment 01<br>Primary reason for amendment:<br>- Changes in section „hypothesis and aims objectives“ regarding primary feasibility outcomes, primary efficacy outcomes and secondary efficacy outcomes.<br>- Changes in „sample section“ regarding sample size calculation, enrollment phase, type of randomisation, retention and attendance rate.<br>- Changes in „statistical analysis“ regarding confidence interval estimation, feasibility and practicability testing,<br>- Changes in „discussion section“ regarding progression criteria<br>Additional changes:<br>- Changes regarding the probiotic intake as supplement |
| Funding | 4 | MVZ Institute of Microecology, Herborn, Germany |
| Roles and responsibilities | 5a | Laura Schmitz1, Nina Ferrari1,5, Andreas Schwiertz2, Kerstin Rusch2, Ulrich Woestmann3 , Esther Mahabir4, Christine Graf1<br><br>[1]Institute of Movement and Neuroscience, Department of Movement and Health Promotion, Am Sportpark Müngersdorf 6, German Sport University Cologne, Cologne, Germany<br><br>[2]MVZ Institute of Microecology, Auf den Lueppen 8, Herborn, Germany<br><br>[3] Academic postgraduate training practice of the University Düsseldorf, Düsseldorf, Germany.<br><br>[4]Comparative Medicine, Center for Molecular Medicine/CMMC Animal Facility, University of Cologne, Robert-Koch-Str. 21, Cologne, Germany<br><br>[5]Cologne Center for Prevention in Childhood and Youth/ Heart Center Cologne, University Hospital of Cologne, Kerpener Str. 62, Cologne, Germany<br><br>Study concept: CG, LS, NF, AS, UW, KR; data collection: LS, CG; data handling: LS, CG, AS, KR, EM; laboratory analyses: LS, AS, KR, EM; data analyses: LS, CG; interpretation of data: LS, CG; preparation of manuscript: LS, CG; critical manuscript revision: NF, AS, EM, CG |
| | 5b | Trial Sponsor: MVZ Institute of Microecology<br>Contact name: Mrs. Dr. med. Kerstin Rusch,<br>Adress: Auf den Lüppen 8, 35745 Herborn, Germany. |
| | 5c | The funding source will have no influence regarding the design of this study and will not have any role during the execution, analyses, interpretation of the data, or decision to submit results. |

5d Organisational structure and responsibility

Complete execution of the study including the following issues: Recruitment of participants, data collection (anthropometric data, food diaries, execution of exercise training, blood and saliva measurements), dataset entry, data analysis: Institute of Movement and Neuroscience, Department of Movement and Health Promotion, Am Sportpark Müngersdorf 6, German Sport University Cologne, Cologne, Germany.

Fecal sample material and microbiota analysis/ diagnostics will be realised by the MVZ Institute of Microecology, Auf den Lueppen 8, Herborn, Germany.

Storage and analysis of blood and saliva samples will be implemented in the Comparative Medicine, Center for Molecular Medicine/CMMC Animal Facility, University of Cologne, Robert-Koch-Str. 21, Cologne, Germany

Preparation of protocol and revisions:

Institute of Movement and Neuroscience, Department of Movement and Health Promotion (CG, NF, LS)

MVZ Institute of Microecology, Auf den Lueppen 8, Herborn, Germany (KR, AS)

Comparative Medicine, Center for Molecular Medicine/CMMC Animal Facility, University of Cologne (EM)

## Introduction

Background and rationale 6a

The intestinal microbiota composition plays a significant role in metabolism and immunity [1, 2]. Certain species and their associated metabolic products influence physiological homeostasis. Metabolic and immune functions include degradation of food, synthesis of vitamins and bioactive compounds/ hormones (e.g. serotonin), training of the immune system, pathogen defense and proliferation of intestinal cells [3, 4]. There is no complete definition of an intact microbiota; however, the following are believed to contribute: an enriched bacterial diversity (alpha diversity), a balanced *Firmicutes/ Bacteroidetes* ratio, an intact mucus layer and an abundance of certain species (e.g. *Akkermansia muciniphila* and *Faecalibacterium prausnitzii*) [5-9]. An altered microbiota contributes to the pathogenesis of diseases such as metabolic or (auto)-immune diseases [10-13]. Recent studies have highlighted that environment and lifestyle factors affect microbiota composition and its metabolic capacity [14, 15]. There is a growing body of evidence indicating that physical activity is linked to specific markers of intestinal health [16-19].

Several studies determined a higher species richness (α diversity) in elite athletes and individuals with higher cardiorespiratory fitness ($VO_2$peak) or higher training frequency than those with a sedentary lifestyle or lower fitness level [16, 17, 20].

Furthermore, an increase in number of the two commensal species *Akkermansia muciniphila* and *Faecalibacterium prausnitzii* was observed in athletes and highly active individuals [17, 18, 20]. This was related to a higher concentration of the health-promoting bacterial metabolite butyrate [18, 20].

Some professional athletes suffer from immunosuppression or gastrointestinal symptoms, including abdominal pain, diarrhea or leaky gut syndrome. This is because excessive exercise reduces intestinal blood flow due to increased circulation in the strained muscles and heart. This may lead to microbial imbalances and mucosa disruption [19, 21-24]. An increased permeability of the intestinal mucosa and epithelium (intestinal barrier) has been associated with bacterial/ pathogen translocation to extraintestinal organs manifesting as intestinal or systemic inflammation [25-27].

Many athletes use probiotics to enhance barrier integrity and to improve gastrointestinal symptoms. Probiotic consumption as supplement may induce immune processes, including the synthesis of cytokines and immunoglobulins, thereby contributing to host health [28-30]. Previous research on trained athletes revealed the beneficial effects of probiotic intake on the marker for mucosal integrity zonulin and several cytokines. Interferon-gamma, tumor necrosis factor-alpha and interleukins were all improved [19, 29, 31-33].

Regular moderate exercise is well-known for its anti-inflammatory efficacy, but diet-driven effects can obscure the intestinal impact of sports per se [17, 18]. Data that determines causality between microbiota composition, mucosa permeability and moderate endurance training are sparse. Longitudinal studies are needed to determine how the intestinal microbiota and mucosa as well as immune markers and cytokines vary with exercise as compared to a probiotic supplementation. A combination of exercise and probiotics may be a new method for the prevention and therapy of intestinal or immune-related diseases [34-36].

[1]Brestoff JR, Artis D. Commensal bacteria at the interface of host metabolism and the immune system. Nature immunology. 2013;14(7):676–84.

[2]Balducci S, et al. Anti-inflammatory effect of exercise training in subjects with type 2 diabetes and the metabolic syndrome is dependent on exercise modalities and independent of weight loss. Nutrition, metabolism, and cardiovascular diseases : NMCD. 2010;20(8):608–17.

[3]Guarner F, Malagelada J-R. Gut flora in health and disease. Lancet (London, England). 2003;361(9356):512–9.

[4]Sekirov I, et al. Gut microbiota in health and disease. Physiological reviews. 2010;90(3): 859–904.

[5]Eckburg PB, et al. Diversity of the Human Intestinal Microbial Flora. Science (New York, NY). 2005;308(5728):1635–8.
[6]Farhadi A, et al. Intestinal barrier: An interface between health and disease. Journal of Gastroenterology and Hepatology. 2003;18(5):479–97.
[7]Ley RE, Peterson DA, Gordon JI. Ecological and evolutionary forces shaping microbial diversity in the human intestine. Cell. 2006;124(4):837–48.
[8]Dao MC, et al. Akkermansia muciniphila and improved metabolic health during a dietary intervention in obesity: relationship with gut microbiome richness and ecology. Gut. 2016;65(3):426–36.
[9]Miquel S, et al. Faecalibacterium prausnitzii and human intestinal health. Current opinion in microbiology. 2013;16(3):255–61.
[10]Serino M, et al. Intestinal microflora and metabolic diseases. Diabetes & metabolism. 2009;35(4):262–72.
[11]Forbes JD, van Domselaar G, Bernstein CN. The Gut Microbiota in Immune-Mediated Inflammatory Diseases. Frontiers in microbiology. 2016;7:1081.
[12]Kassinen A, et al. The fecal microbiota of irritable bowel syndrome patients differs significantly from that of healthy subjects. Gastroenterology. 2007;133(1):24–33.
[13]Macfarlane GT, et al. The gut microbiota in inflammatory bowel disease. Current pharmaceutical design. 2009;15(13):1528–36.
[14]Conlon MA, Bird AR. The impact of diet and lifestyle on gut microbiota and human health. Nutrients. 2014;7(1):17–44.
[15]Monda V, et al. Exercise Modifies the Gut Microbiota with Positive Health Effects. Oxidative medicine and cellular longevity. 2017;2017:3831972.
[16]Estaki M, et al. Cardiorespiratory fitness as a predictor of intestinal microbial diversity and distinct metagenomic functions. Microbiome. 2016;4.
[17]Clarke SF, et al. Exercise and associated dietary extremes impact on gut microbial diversity. Gut. 2014;63(12):1913–20.
[18]Bressa C, et al. Differences in gut microbiota profile between women with active lifestyle and sedentary women. PloS one. 2017;12(2):e0171352.
[19]Lamprecht M, et al. Probiotic supplementation affects markers of intestinal barrier, oxidation, and inflammation in trained men; a randomized, double-blinded, placebo-controlled trial. Journal of the International Society of Sports Nutrition. 2012;9:45.
[20]McFadzean R. Exercise can help modulate human gut microbiota2014.
[21]Lachtermann E, Jung K. Sport und gastrointestinales System - Einfluss und Wechselwirkungen. Deutsches Ärzteblatt. 2006(103):2116–20.
[22]Pedersen BK. Special feature for the Olympics: effects of exercise on the immune system: exercise and cytokines. Immunology and cell biology. 2000;78(5):532–5.
[23]Petersen AMW, Pedersen BK. The role of IL-6 in mediating the anti-inflammatory effects of exercise. Journal of physiology and pharmacology : an official journal of the Polish Physiological Society. 2006;57 Suppl 10:43–51.
[24]Oliveira EPd, Burini RC, Jeukendrup A. Gastrointestinal complaints during exercise: prevalence, etiology, and nutritional recommendations. Sports medicine (Auckland, NZ). 2014;44 Suppl 1:S79-85.
[25]Fasano A. Zonulin and its regulation of intestinal barrier function: the biological door to inflammation, autoimmunity, and cancer. Physiological reviews. 2011;91(1):151–75.
[26]Alonso C, et al. Intestinal barrier function and the brain-gut axis. Advances in experimental medicine and biology. 2014;817:73–113.
[27]Baumgart DC, Dignass AU. Intestinal barrier function. Current opinion in clinical nutrition and metabolic care. 2002;5(6):685–94.
[28]Pyne DB, et al. Probiotics supplementation for athletes - clinical and physiological effects. European journal of sport science. 2015;15(1):63–72.
[29]Cox A, Pyne, DB, Saunders, PU, Fricker, PA. Oral administration of the probiotic Lactobacillus fermentum VRI-003 and mucosal immunity in endurance athletes. Br J Sports Med. 2010;44:222–6.
[30]Rescigno M. Intestinal microbiota and its effects on the immune system. Cellular microbiology. 2014;16(7):1004–13.
[31]Kekkonen RA, et al. The Effect of Probiotics on Respiratory Infections and Gastrointestinal Symptoms during Training in Marathon Runners. International journal of sport nutrition and exercise metabolism. 2007;17(4):352–63.
[32]Gleeson M, et al. The anti-inflammatory effects of exercise: mechanisms and implications for the prevention and treatment of disease. Nature reviews Immunology. 2011;11(9):607–15.
[33]Gleeson M, et al. Effects of a Lactobacillus salivarius probiotic intervention on infection, cold symptom duration and severity, and mucosal immunity in endurance athletes. International journal of sport nutrition and exercise metabolism. 2012;22(4):235–42.
[34]McFarland LV. Meta-analysis of probiotics for the prevention of antibiotic associated diarrhea and the treatment of Clostridium difficile disease. The American journal of gastroenterology. 2006;101(4):812–22.
[35]West CE. Probiotics for allergy prevention. Beneficial Microbes. 2016;7(2):171–9.
[36]Ukena SN, et al. Probiotic Escherichia coli Nissle 1917 inhibits leaky gut by enhancing mucosal integrity. PloS one. 2007;2(12):e1308.

| | | |
|---|---|---|
| | 6b | Choice of comparators<br>In this controlled cross-over trial we will compare the potential influence of regular exercise training and probiotic supplement SymbioLactComp®<br><br>Training intensity uses the baseline shuttle run results (heart rate peak). Intensity of exercises will increase from 70% to 85% of the heart rate peak to improve the subject's basic endurance. The three 60-minute trainings per week will consist of extensive and intensive continuous methods, interval training and stretching.<br>The probiotic period involves supplementation with the probiotic SymbioLactComp® (SymbioPharm GmbH, Herborn, Germany). SymbioLactComp® contains different lactic acid bacteria (*Lactobacillus paracasei*, *Lactobacillus acidophilus*, *Lactococcus lactis* and *Bifidobacterium animalis subsp. lactis*) as well as 30 µg of biotin. The total bacteria count of one sachet (2g) is ≥1 x $10^9$ KBE/g. It will be mixed with water and taken daily at breakfast. |
| Objectives | 7 | **Research Hypothesis**<br><br>We hypothesize that a training period with regular running exercises may alter microbiota composition in healthy adults. Participants receiving the intervention might further show higher rates of selected markers of intestinal health, like Akkermansia muciniphila, Faecalibacterium prausnitzii, Bifidobacterium spp. and lower rates of intestinal immune markers; e.g. zonulin, alpha-1-antitrypsin or secretory immunoglobulin A (sIgA).<br><br>**Aims and study objectives**<br><br>See below at „outcomes". |
| Trial design | 8 | This randomised, controlled, cross-over trial is conducted at the German Sport University Cologne in cooperation with the MVZ Institute of Microecology GmbH. |

**Methods: Participants, interventions, and outcomes**

| | | |
|---|---|---|
| Study setting | 9 | Data will be collected through bachelor endurance courses at the German Sport University Cologne, Cologne, Germany. |
| Eligibility criteria | 10 | Inclusion criteria<br>Age between 18 and 29 and a body mass index of ≤ 25 kg/m$^2$ .<br>Exclusion criteria<br>Injuries, diseases (e.g. inflammatory bowel diseases), an antibiotic therapy just before or during the intervention and lasting gastrointestinal disturbances based on probiotic intake. |

| | | |
|---|---|---|
| Interventions | 11a | Participants will receive a twelve-week intervention program including a two-week rest, a four-week endurance training period and a four-week probiotic period with SymbioLactComp®. Subjects will not perform endurance sports or take probiotics during the rest period, but will participate in the university courses during the program.<br><br>Training intensity uses baseline shuttle run results (HRpeak). The training goal is a 10km-run in 50 minutes (males) or 55 minutes (female). The intensity of exercises will increase from 70% to 85% of the HRpeak to improve the subject's basic endurance [39]. The three 60-minute trainings per week will consist of extensive and intensive continuous methods, interval training and stretching [39]. The exercises will be performed in groups and supervised by a sports scientist. Sensitivities will be checked using a Borg Scale; the heart rate will be measured by a pulse monitor and belt from Polar® (Polar® Typ FT1). Participants will keep a training diary to log the completion of exercises. A training plan is shown in more detail.<br><br>The probiotic period involves supplementation with the probiotic SymbioLactComp® (SymbioPharm GmbH, Herborn, Germany). SymbioLactComp® contains different lactic acid bacteria (*Lactobacillus paracasei*, *Lactobacillus acidophilus*, *Lactococcus lactis* and *Bifidobacterium animalis subsp. lactis*) as well as 30 µg of biotin. The total bacteria count of one sachet (2g) is ≥1 x $10^9$ KBE/g. It will be mixed with water and taken daily at breakfast.<br><br>Venous blood samples (of approximately 25 mL) and saliva samples will be collected under medical supervision. Fecal samples and nutrition diary will be collected at everyones' home. |
| | 11b | Criteria for discontinuing or modifying allocated interventions for a given trial participant are the following:<br>Antibiotic therapy, injuries, pain, headache, other complaints that do not allow participation in the (sports) program. In addition, meteorism, diarrhea or other gastrointestinal complaints as side effects of taking probiotics, which also do not allow participation in the program. In case of appropriate symptoms, participants will take a break or cancel participation, if necessary after consultation with the study management and the attending physician. |

| | | |
|---|---|---|
| | 11c | Adherence<br><br>Participants will keep a training diary as well as a food diary to improve adherence of the intervention program.<br>Participants will get instructions of taking the probiotic supplement and collecting fecal samples as well as the shipping of the samples. |
| | 11d | Relevant concomitant care and interventions during the trial:<br>- Use of borg scale and pulse watch to determine the participants' physical load during the training.<br>- Medical consultation during the probiotic period, if probiotic induced complaints will arise.<br>- Medical supervision during the blood and saliva measurements |
| Outcomes | 12 | Primary feasibility outcomes of this randomised control cross-over trial are to assess practicability of the intervention (physical activity and probiotic supplementation) and capability of study design as well as to provide reliable estimates for sample size calculation. Primary efficacy outcome is the potential influence of moderate endurance training on the intestinal microbiota (microbiota composition) and barrier function (selected mucosa and immune parameters) in healthy student athletes compared to a probiotic supplement (SymbioLactComp®). Secondary efficacy outcomes will be selected blood and saliva biomarkers (haemogram, selected cytokines). |

| | | |
|---|---|---|
| Participant timeline | 13 | The study will have a half-year enrollment phase. Participants will be recruited through bachelor endurance courses (maximal participant rate around 320 students) within one semester. The planned period for the whole intervention is for a maximum of one year.<br>Schematic of the study design: |

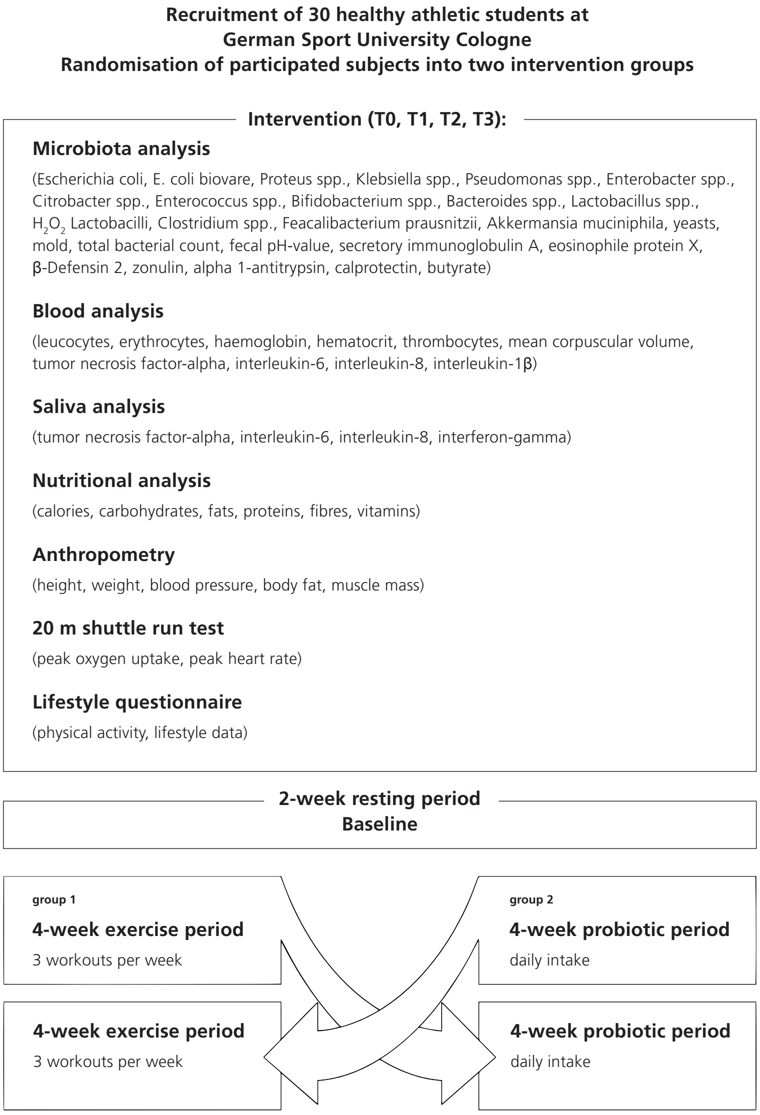


Additional file shows study design

| | | |
|---|---|---|
| Sample size | 14 | Sample size is calculated to recruit at least 30 participants in order to estimate the pooled standard deviation of the outcome with a reasonable degree of precision for a future definitive trial (Sim et al., 2011, Hayman et al., 2012). Healthy students with an age between 18 and 29 and a body mass index of ≤ 25 $kg/m^2$ will be recruited through bachelor endurance courses at the German Sport University Cologne (maximal participant rate around 320 students). Therefore, our feasibility study enrollment rate has to exceed 10%. Participants will be randomised in a 1:1 ratio and matched to group one (endurance-probiotics) or group two (probiotics-endurance) via block randomisation (Suresh et al., 2011).<br><br>Sim J, Lewis M. The size of a pilot study for a clinical trial should be calculated in relation to considerations of precision and efficacy. J Clin Epidemol. 2012;65(3): 301-8.<br>Hayman A, et al., How big should a pilot trial be? A case for continuous outcomes in Randomized Controlled Trials. Annual Conference of the Royal Statistical Society; Telford; UK2012.<br>Suresh K. An overview of randomization techniques: An unbiased assessment of outcome in clinical research. Journal of human reproductive sciences. 2011;4(1): 8-11. |
| Recruitment | 15 | Potential student athletes will be achieved via flyers and short talks in the bachelor endurance courses at the German University Cologne. Interested students will receive contact data from the study administration and will be invited to a personal meeting with the study director. |

**Methods: Assignment of interventions (for controlled trials)**

Allocation:

| | | |
|---|---|---|
| Sequence generation | 16a | Key elements of randomisation:<br>method: random number table<br>Type of randomisation: blocked randomisation<br>Participants will be randomised in a 1:1 ratio and matched to group one (endurance-probiotics) or group two (probiotics-endurance). |
| Allocation concealment mechanism | 16b | |
| Implementation | 16c | All patients who give consent for participation and who fulfil the inclusion criteria will be randomised. Randomisation will be requested by the study director. |

| | | |
|---|---|---|
| Blinding (masking) | 17a | |
| | 17b | |

**Methods: Data collection, management, and analysis**

| | | |
|---|---|---|
| Data collection methods | 18a | A standardised multistage 20 m shuttle run test will be performed at the outset of the study, measuring Heart rate peak and the $VO_2$peak as an indicator for the participants' cardiorespiratory fitness.<br>Participants will complete four detailed seven-day food diaries based on self-reported intake. A standardised protocol will be developed by a research dietitan supervisor.<br>Furthermore, a personal history and lifestyle including physical activity, will be collected by a modified questionnaire (International Physical Activity Questionnaire at baseline.<br><br>Venous blood samples will be collected using the BD Vacutainer® Butterfly Safety Lok, EDTA tubes and SST™ II Advance serum tubes (Becton Dickinson, Heidelberg, Germany). The thawed serum samples will be used to measure the following cytokines: tumor necrosis factor-alpha, interleukin-6, interleukin-8, interleukin-1β by multiplex ELISA (Bio-Plex Pro Human Cytokine 8-plex ELISA Kit, Bio-Plex Pro Human Chemokine IL-1β Singleplex Set; Bio-Rad Laboratories GmbH, Munich, Germany).<br><br>Saliva samples will be collected after a 30-minute fast via Salivettes® from Sarstedt (Sarstedt AG & Co. KG, Nümbrecht, Germany). Selected cytokines will be analysed: tumor necrosis factor-alpha, interleukin-6, interleukin-8, interferon-gamma by a multiplex ELISA (Bio-Plex Pro Human Cytokine 8-plex, Bio-Rad Laboratories GmbH, Munich, Germany).<br><br>Fecal analyses will be carried out at the Institute of Microoecology, Herborn, Germany. Bacteria will be enumerated using the KyberKompaktPro® test, which combines the identification of viable bacteria by classical microbial analysis and quantitative polymerase chain reaction (qPCR). Human stool samples analysed for SCFA content will be freeze-dried and subsequently analysed using a gas chromatograph. Fecal calprotectin, eosinophil protein x (EPX), zonulin, beta-defensin 2 (β-defensin 2) and secretory immunoglobulin A (sIgA) concentrations will be measured by an ELISA kit (Immundiagnostik AG, Bensheim, Germany). |

| | | |
|---|---|---|
| | 18b | Retention and attendance rates will be assessed to calculate adherence. The retention rate will be analysed by the percentage of participants completing all sessions of the endurance training (twelve in total), shuttle run test (two in total), nutrition questionnaire (four in total), blood/saliva measurement (four in total) and stool diagnostics (four in total) present in each step of study. Attendance rate will be analysed by the mean of presences of the participants divided by the number of sessions offered during the four-week exercise period and the four-week probiotic period. |
| Data management | 19 | All data will be entered electronically at the Institute of Movement and Neuroscience, Department of Movement and Health Promotion, German Sport University Cologne.<br>Participant files will be stored in a secure and accessible place and will be kept in locked cabinets. Access to the study data will be restricted.<br>Data protection will be guaranteed through a password system. |

| | | |
|---|---|---|
| Statistical methods | 20a | This pilot study intends to examine the confidence interval estimation, feasibility and practicability of the presented approach (physical activity and probiotic supplementation) (Leon et al., 2011, Arain et al. 2004). Our trial is mainly conducted in preparation for a future definitive randomised controlled trial to assess efficacy of the intervention/ study design (*Eldridge et al., 2016*). All data will be entered into the database management software and analysed by the SPSS Version 25.0 (SPSS Inc., Chicago, IL, USA). The Shapiro-Wilk test will be used for variable normal distribution and the usual descriptive statistics for background information and mean values with a 95 % confidence interval. Data will be used to determine the most appropriate outcome measure for the main study and in sample size estimation. Descriptive statistics and confidence intervals will be also carried out to examine the trends in the analysed parameters. Depending on the normality of the underlying data, parametric or non-parametric (e.g. unpaired t test/ Mann-Whitney-U test) tests will be carried out. The level of statistical significance is $p < 0.05$.<br><br>Leon AC, Davis LL, Kraemer HC. The role and interpretation of pilot studies in clinical research. J Psychiatr Res. 2011;45(5): 626-9.<br>Arain M, et al., What is a pilot or feasibility study? A review of current practice and editorial policy. BMC Med Red Methodol. 2010;10:67.<br>Eldridge SM, et al., CONSORT 2010 statement: extension to randomised pilot and feasibility trials. Pilot Feasibility Stud. 2016;2:64. |
| | 20b | |
| | 20c | All randomised participants, regardless of protocol adherence will be included in the electronical dataset. Missing data will be marked by using the variable 999. |
| **Methods: Monitoring** | | |
| Data monitoring | 21a | A data monitoring committee (DMC) is not needed because of the short duration as well as the known minimal risk of the intervention program (endurance training and probiotic intake). |
| | 21b | |
| Harms | 22 | The study will monitor for the following adverse effects concerning the endurance training and probiotic supplementation:<br>Muscle injuries, intolerance towards the probiotic SymbioLact. |

| | | |
|---|---|---|
| Auditing | 23 | The study director will regularly review the dataset to determine whether the data reported are complete and accurate. |

**Ethics and dissemination**

| | | |
|---|---|---|
| Research ethics approval | 24 | The study was approved by the ethic committee of the German Sport University Cologne (No. 136/2016). |
| Protocol amendments | 25 | Any amendments will be transparently described in trial reports. |
| Consent or assent | 26a | Subjects who meet the inclusion criteria, will obtain detailed information about the trial and will give written informed consent to the study director before starting the study. |
| | 26b | |
| Confidentiality | 27 | Participants' data will not be released outside of the study without the written permission of the participant. Furthermore, all study-related data will be stored securely in locked file cabinets in a room with limited access. Laboratory data will be identified by a coded Identification number only to maintain participant confidentially. |
| Declaration of interests | 28 | The authors declared that they have no conflict of interests. |
| Access to data | 29 | The study director (Laura Schmitz) and the medical director (Christine Graf) as well as the representative study director (Nina Ferrari) will have access to the final dataset of this study. |
| Ancillary and post-trial care | 30 | The attending physician ensures that this study is conducted in accordance with the Declaration of Helsinki (additions to Tokyo, Venice and Hong Kong). |
| Dissemination policy | 31a | The time between the completion of data collection and evaluation and the release of the study results will be reduce to a minimum. The trial results will be released to the participants as well as published, regardless of the statistical significance. |
| | 31b | Only individuals who meet the criteria for authorship will be listed as authors. There won't make use of professional writers or of ghost authorship. |
| | 31c | We will deliver a completely deidentified data set after the collection of all data. |

**Appendices**

Informed consent materials

32

Einwilligungserklärung

Name: ______________________________________________

Geburtsdatum: ______________________________________________

Das Original dieser Einwilligungserklärung verbleibt bei den Unterlagen. Eine Kopie der Einwilligungserklärung wird den Teilnehmern ausgehändigt.

Ich ______________________________________________

(Vorname, Name)

erkläre, dass ich das Informationsblatt zur wissenschaftlichen Untersuchung:

„Zusammenhang von Darmmikrobiota und Sport“

und diese Einwilligungserklärung erhalten habe.

[?] Ich wurde für mich ausreichend mündlich und schriftlich über die wissenschaftliche Untersuchung informiert.

[?] Ich erkläre, dass ich damit einverstanden bin, dass Stuhl, Blut und Speichel, welches entnommen bzw. abgegeben wird, für die o. g. wissenschaftliche Untersuchung genutzt werden kann.

[?] Ich weiß, dass ich jederzeit meine Einwilligung, ohne Angaben von Gründen, widerrufen kann, ohne dass dies für mich nachteilige Folgen hat. Beim Widerruf meiner Einwilligung, an der Studie teilzunehmen, habe ich das Recht, die Löschung aller meiner bis dahin gespeicherten personenbezogenen Daten sowie eine Vernichtung von personenbezogenen Proben zu verlangen.

[?] Ich bin damit einverstanden, dass die im Rahmen der wissenschaftlichen Untersuchung über mich erhobenen pseudonymisierten Daten sowie meine sonstigen mit dieser Untersuchung zusammenhängenden personenbezogenen Daten aufgezeichnet werden. Es wird gewährleistet, dass meine personenbezogenen Daten nicht an Dritte weitergegeben werden. Bei der Veröffentlichung in einer wissenschaftlichen Zeitung wird aus den Daten nicht hervorgehen, wer an dieser Untersuchung teilgenommen hat. Meine persönlichen Daten unterliegen dem Datenschutzgesetz.

☐ ·Bei Verletzungen, anhaltenden Magen-Darm-Beschwerden, anhaltenden Kopfschmerzen oder sonstigen Anzeichen einer Erkrankung, die eine Teilnahme am (Sport-)Programm nicht ermöglichen, informiere ich umgehend die Studienleitung und hole ggf. eine eingehende fachärztliche Beurteilung ein.

☐ Nebenwirkungen unter der Einnahme von Probiotika sind selten, können aber Blähungen, Durchfall und Unwohlsein bedeuten. Bei entsprechender Beschwerdesymptomatik wird zunächst eine Pause eingelegt, ggf. Nach Rücksprache mit der Studienleitung und dem behandelnden Arzt die Teilnahme abgebrochen. Nebenwirkungen eines mehrwöchigen Ausdauertrainings können durch Fehlbelastungen oder Überbeanspruchung entstehen. Bei Muskel-/Sehnen-/ Gelenkverletzungen, Schmerzen oder Kreislaufproblemen wird ebenfalls zunächst pausiert, ggf. Nach Rücksprache mit der Studienleitung und dem behandelnden Arzt die Teilnahme abgebrochen.

☐ ·Mit der vorstehend geschilderten Vorgehensweise bin ich einverstanden und bestätige dies mit meiner Unterschrift.

Datum, Ort, Unterschrift Teilnehmer/in

| | | |
|---|---|---|
| Biological specimens | 33 | Aliquots with blood and saliva as well as fecal samples will be bar coded with a unique storage identifier.<br>The scientists who will carry out blood, saliva and fecal analyses will not have access to personal identifiers and will not be able to link the results of the tests to personal identifier information. No individual results will be presented in publications. |

*It is strongly recommended that this checklist be read in conjunction with the SPIRIT 2013 Explanation & Elaboration for important clarification on the items. Amendments to the protocol should be tracked and dated. The SPIRIT checklist is copyrighted by the SPIRIT Group under the Creative Commons "Attribution-NonCommercial-NoDerivs 3.0 Unported" license.
