# Supplementary material for: Impact of endurance exercise and probiotic supplementation on the intestinal microbiota: a cross-over pilot study
Source: Pilot Feasibility Stud. 2019 Jun 8;5:76. doi: 10.1186/s40814-019-0459-9 (PMC6556026; doi:10.1186/s40814-019-0459-9)
Supplement: Supplementary file 3 — Training plan (PDF 185 kb) [file 40814_2019_459_MOESM3_ESM.pdf]

Each workout starts with warm up (10-15 min ABC running form drills) and ends with stretching (10 min)

		Content	Intensity/Peak heart rate
**First training week**			
	First workout	2 x 1000 m target speed<br><br>4 km moderate endurance run	Ca. 75-85% HRpeak<br><br>Ca. 70-75% HRpeak
	Second workout	5 x 400 m interval<br><br>3 min breaks between intervals<br><br>15 min stabilisation training	Ca. 80-85% HRpeak<br><br>Ca. 70% HRpeak<br><br>/
	Third workout	6 km run	Ca. 70-75% HRpeak
**Second training week**			
	First workout	3 x 1000 m target speed<br><br>5 km moderate endurance run	Ca. 75-85% HRpeak<br><br>Ca. 70-75% HRpeak
	Second workout	6 km endurance run at aerobic threshold	Ca. 75-85% HRpeak
	Third workout	8 km run	Ca. 70-80% HRpeak
**Third training week**			
	First workout	2 x 2000 m target speed<br><br>6 km moderate endurance run	Ca. 75-85% HRpeak<br><br>Ca. 70-75% HRpeak
	Second workout	6 x 400 m interval<br><br>3 min breaks between intervals<br><br>15 min stabilisation training	Ca. 75-85% HRpeak<br><br>Ca. 70% HRpeak<br><br>/
	Third workout	8 km endurance run	Ca. 75-85% HRpeak
**Fourth training week**			
	First workout	3 x 2000 m target speed<br><br>6 km moderate endurance run	Ca. 75-85% HRpeak<br><br>Ca. 70-75% HRpeak
	Second workout	7 x 400 m interval<br><br>3 min breaks between intervals<br><br>15 min stabilisation training	Ca. 75-85% HRpeak<br><br>Ca. 70% HRpeak<br><br>/
	Third workout	10 km run	Ca. 75-85% HRpeak

Additional file 2 shows exercise period
